# Residues of Desiccant Herbicides in Sesame Seeds (*Sesamum indicum* L.) Following Preharvest Application Determined by LC-MS/MS

**DOI:** 10.1021/acs.jafc.6c06511

**Published:** 2026-07-01

**Authors:** Laura Bordignon, Rodrigo Monte Lorenzoni, Thiago Svacina, Rodrigo Floriano Pimpinato, Kassio Ferreira Mendes

**Affiliations:** † Center for Nuclear Energy in Agriculture, 28133 University of São Paulo, Centenário Avenue 303, São Dimas, 13400-970 Piracicaba, São Paulo, Brazil; ‡ Research and Development Department, 28120 Sebra Agrícola LTDA, Lot. Canarana I, Chácara G8, 78640-000 Canarana, Mato Grosso, Brazil

**Keywords:** desiccation, safe foods, pesticide residues, maximum residue limits

## Abstract

As global sesame (*Sesamum indicum* L.) exports expand, compliance with international Maximum Residue Limits (MRLs) is essential. This study evaluated the effects of different desiccation rates of diquat, saflufenacil, and glyphosate, and its metabolite AMPA, on residue levels in sesame seeds. Desiccation was performed 85 days after planting, and harvest was conducted 10 days after application for diquat and 20 days after application for glyphosate and saflufenacil. Herbicide residues were subsequently determined by LC-MS/MS. Diquat and saflufenacil residues remained below the limit of quantification (LoQ) in all treatments. However, neither herbicide provided satisfactory desiccation, as diquat caused rapid necrosis and seed burning, while saflufenacil showed limited efficacy. Glyphosate promoted slower desiccation with substantial residue accumulation in sesame seeds (23.6–51.7 mg kg^–1^), exceeding established MRLs, whereas AMPA remained below the LoQ. These findings highlight the need for crop-specific recommendations and residue monitoring programs to support safe sesame production and international trade.

## Graphical abstract

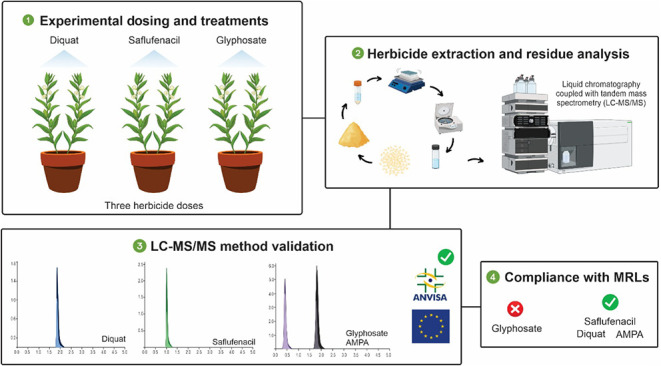

## 1 Introduction

Sesame cultivation has recently expanded in Brazil due to its advantageous agronomic traits, low production costs and increasing global demand, making it an attractive alternative crop for off-season rotation systems, particularly after soybean, cotton, or maize.^1,2^ This expansion has positioned Brazil as a significant producer and exporter.^3^

Despite the rapid expansion and strong commercial performance, sesame cultivation in Brazil still encounters several technical and regulatory challenges. Its indeterminate growth habit leads to asynchronous flowering and capsule maturation, which complicates harvest timing and increases susceptibility to yield losses.^4^ Frequent rainfall during ripening further aggravates these losses by increasing seed moisture and inducing pod cracking.^5^ To address these challenges, desiccant herbicides have been proposed to accelerate and standardize plant drying, facilitating mechanical harvest.^6,7^

Despite these advantages, no desiccants are officially registered for sesame cultivation in Brazil.^8^ As sesame is not a major crop in leading agricultural nations, pesticide companies have limited incentive to develop herbicides specifically for this crop.^9^ This regulatory gap remains a major limitation to improving yield and expanding production, often leading to the off-label use of unapproved products without crop-specific guidelines such as defined preharvest intervals (PHIs) and maximum residue limits (MRLs).^2,10^

The lack of authorized chemical options not only constrains weed and harvest management but also increases the risk of pesticide residues in seeds at harvest, potentially compromising food safety and market access.^11^ Desiccant herbicides are applied only a few days before harvest, making the absence of specific PHIs particularly critical. Therefore, defining PHI guidelines is essential to ensure an adequate time gap between the application and the harvest, preventing noncompliance.^10^ Consequently, the establishment of safe, crop-specific, and effective desiccation protocols is crucial to ensuring production efficiency and regulatory compliance.

Pesticide residues in food have become a growing global concern for consumers and regulatory agencies worldwide.^12^ Establishing and monitoring MRLs is essential to ensure that pesticide concentrations in food remain within safe limits while supporting fair trade practices.^13^ Although sesame has gained economic importance, only a few studies have analyzed pesticide residues in sesame seeds, and most focus on a limited range of compounds.^11^ Continuous residue monitoring in this crop is essential not only for protecting public health but also for guaranteeing product quality and traceability, thereby improving agricultural management practices.^10^

Given the growing demand for safe food and increasing concerns regarding pesticide residues,^14^ there is a pressing need for traceability studies that assess the persistence and behavior of pesticide residues in sesame grains. Therefore, this study aimed to evaluate the traceability of residues of the desiccant herbicides diquat, saflufenacil and glyphosate and the metabolite aminomethylphosphonic acid (AMPA) in sesame grains by liquid chromatography coupled to tandem mass spectrometry (LC-MS/MS), identify potential compliance risks, and enhance agricultural practices, residue monitoring, and quality assurance systems in Brazilian sesame production.

## 2 Materials and Methods

### 2.1 Experimental Design and Treatments

The experiment was conducted in a completely randomized design arranged in a 3 × 3 factorial scheme with four replicates, aiming to evaluate the effects of different herbicides applied at varying doses. The factors consisted of three herbicides (diquat, glyphosate, and saflufenacil) and three spraying rates.

### 2.2 Materials

The analytical standards of the herbicides spiked into the sesame samples had a chemical purity of 99% (w w^–1^). The analytical standards and solvents used for chromatographic elution (formic acid, acetic acid, and acetonitrile) were purchased from Sigma-Aldrich (San Luis, MO, USA). Deionized and ultrapure water were obtained from a reverse osmosis purification system (Millipore, Milli-Q Direct 8 UV with Pump).

For herbicide extraction, magnesium sulfate, sodium chloride, dichloromethane, and methanol were purchased from Merck (Merck KGaA, Darmstadt, Germany), while C_18_ and PSA sorbents were obtained from Agilent Technologies (Santa Clara, CA, USA). Filtration was performed using 0.22 μm regenerated cellulose membrane filter (Millipore, PTFE membrane).

The commercial herbicide formulations used were Diquat Nortox (200 g a.e. L^–1^), Roundup Original Mais (diammonium salt, 480 g a.e. L^–1^) and Heat (700 g a.i. kg^–1^), all obtained from local suppliers.

### 2.3 Sesame Cultivation, Herbicide Spraying, and Desiccation Analyses

Sesame plants were grown in 5.5 L pots containing two plants each. The physicochemical properties of the soil used for cultivation are provided in the Supporting Information (Table S1). Herbicide applications were performed 85 days after planting using a CO_2_-pressurized backpack sprayer equipped with a TTI 110.02 nozzle tip, providing a spray width of 0.5 m. The spray volume was 200 L ha^–1^, applied at a pressure of 200 kPa and a travel speed of 1.0 m s^–1^.

The application doses for each herbicide were established based on the recommended desiccation rates for other crops, since no label information is currently available for sesame. Diquat doses were based on soybean desiccation rates, glyphosate on black oat, and saflufenacil on cotton. The applied rates were 200, 300, and 400 g a.e. ha^–1^ for diquat, 35, 52.5, and 70 g a.i. ha^–1^ for saflufenacil, and 480, 720, and 960 g a.e. ha^–1^ for glyphosate. Desiccation efficiency was evaluated qualitatively through visual observations of plant drying, pod appearance, and symptom progression after herbicide application, following practical desiccation criteria commonly adopted by sesame producers under commercial cultivation conditions.

Plants treated with diquat were harvested 10 days after application (DAA), whereas those treated with glyphosate and saflufenacil were collected at 20 DAA. Harvest was delayed until the maximum feasible time to allow plant drying. After harvest, plants that still exhibited partially green tissues were oven-dried at 45 °C for 24 h, to standardize moisture content. Subsequently, the seeds were ground under liquid nitrogen and stored frozen until residue analysis by LC-MS/MS (Agilent Technologies, 6410 Triple Quad LC/MS, Santa Clara, CA, USA).

### 2.4 LC-MS/MS Conditions and Analyses

The LC-MS/MS system was equipped with an electrospray ionization (ESI) source. The capillary voltage was maintained at ±3000 V, and the source heater temperature was set to 280 °C. Nitrogen was used as the nebulizing and drying gas at a flow rate of 12 L min^–1^ and a pressure of 60 psi. The multiple reaction monitoring (MRM) parameters used for the detection of diquat, saflufenacil, glyphosate, and AMPA are detailed in Table 1. Data acquisition and instrument control were performed using MassHunter software (version B.04.00).

**Table 1:** Multiple Reaction Monitoring (MRM) Parameters for the Detection of Diquat, Saflufenacil, Glyphosate, and Its Metabolite AMPA by LC-MS/MS

Compound	Precursor ion (*m*/*z*)	Product ion (*m*/*z*)	Dwell (ms)	Fragmentation energy (V)	Collision energy (V)	Acceleration voltage (V)
Diquat	183	157	20	120	24	7
Diquat	183	130	20	120	40	7
Saflufenacil	501.1	459	20	160	8	7
Saflufenacil	501.2	349	20	160	24	7
Glyphosate	168	150	500	135	8	7
Glyphosate	168	63	500	135	24	7
AMPA	110	79.2	250	135	28	7
AMPA	110	63.1	250	135	20	7

Chromatographic separations for diquat and saflufenacil were performed under the same instrumental conditions, using an Agilent Zorbax Eclipse column (150 mm × 2.1 mm × 3.5 μm) maintained at 35 °C. Both analytes were detected in positive ionization mode (ESI^+^). The mobile phases consisted of 0.1% (v v^–1^) formic acid aqueous solution (phase A) and acetonitrile containing 0.1% (v v^–1^) formic acid (phase B), with a flow rate of 0.4 mL min^–1^ under isocratic conditions at a proportion of 5:95 (A:B). Due to the distinct physicochemical properties of diquat and saflufenacil, different extraction procedures were required for each compound, therefore, analyses were carried out separately. The total run time for both analyses was 5 min.

The chromatographic separation of glyphosate and AMPA was performed simultaneously using a Raptor Polar column (Restek, 30 mm × 2.1 mm × 2.7 μm) at 35 °C. Both compounds were analyzed in negative ionization mode (ESI^–^). The mobile phases were composed of 0.5% (v v^–1^) formic acid aqueous solution (phase A) and acetonitrile containing 0.5% (v v^–1^) formic acid (phase B), under a flow rate of 0.4 mL min^–1^. The elution program consisted of an isocratic proportion of 95:5, with a total run time of 5 min.

### 2.5 LC-MS/MS Method Validation

The chromatographic method validation was assessed in accordance with the parameters of selectivity, linearity, recovery (accuracy), precision, and limits of detection (LoD) and quantification (LoQ), as described in RDC No. 4 by Anvisa^15^ and DOQ-CGCRE-008 by Inmetro.^16^

Selectivity was evaluated by comparing chromatograms of sesame extracts spiked with analytical standards of the target herbicides and of the blanks (nonfortified) extracts. This assessment aimed to verify the absence of interfering peaks at the same retention times (Rt) as the analytes of interest.

Linearity was verified through calibration curves constructed with five increasing concentrations of each herbicide, analyzed in triplicate. The coefficient of determination (R^2^) was used as the criterion for linearity assessment, demonstrating the method’s ability to produce analytical responses directly proportional to the analyte concentrations. Blank sesame extracts were subjected to the same extraction procedure as fortified samples and subsequently used to prepare calibration standards. Calibration levels for diquat and saflufenacil were established at 0.1, 0.15, 0.3, 0.5, and 1.0 μg mL^–1^, while for glyphosate and AMPA they were set at 0.10, 0.20, 0.50, 1.00, and 2.00 μg mL^–1^.

Recovery experiments were performed to evaluate the extraction efficiency of the method. Sesame samples were fortified at three levels for each analyte: 0.015, 0.03, and 0.06 for diquat; 0.20, 0.30, and 0.50 for saflufenacil; and 0.20, 0.30, and 0.40 μg mL^–1^ for glyphosate and AMPA. Recovery was determined by establishing the relationship between the amount of analyte used to fortify the sample and the amount quantified by the chromatographic system.

Precision was expressed in terms of repeatability and spiked with the standards using the same levels applied for the recovery assays. It was determined by the relative standard deviation (RSD) of replicate measurements to the mean concentration obtained.

The LoD and LoQ values were determined based on the signal-to-noise ratio of three free samples and the slope of the analytical calibration curve. The LoD was defined as the lowest concentration capable of producing a signal distinguishable from the background noise, corresponding to approximately 3.3 times the standard deviation divided by the slope of the curve. The LoQ was defined as the minimum concentration that can be quantified with acceptable precision and accuracy, corresponding to about 10 times this ratio.

### 2.6 Sample Preparation and Extraction

The extraction procedure for diquat was based on the method proposed by Shinde et al.^11^ For the extraction, 3.0 g of sesame seed ground sample was weighed into 50 mL polypropylene tubes. Subsequently, 5 mL of ultrapure water was added to hydrate the sample, followed by vortex agitation for 30 s. Then, 5 mL of acetonitrile were added, and the mixture was vortexed again for another 30 s. Afterward, 4 g of magnesium sulfate and 1 g of sodium chloride were added, and the tube was vortexed for 2 min. The samples were then centrifuged (Hitachi CF16RXII) at 1500 × *g* for 5 min to promote phase separation. After separation, 1 mL of the clear supernatant was transferred to a 2 mL polypropylene tube for dispersive solid-phase extraction (d-SPE). To each tube, 100 mg of C_18_ and 150 mg of magnesium sulfate were added, and the mixture was vortexed for 30 s and centrifuged at 5040 × *g* for 5 min.

The extraction of saflufenacil from sesame was adapted from Tong et al.^17^ A 2.0 g sample was weighed into a 50 mL polypropylene tube, and 2 mL of ultrapure water were added. Subsequently, 10 mL of acetonitrile containing 1% acetic acid were added, followed by vortex agitation for 1 min. Then, 4 g of magnesium sulfate, 1 g of sodium chloride, and a ceramic homogenization stone were added, and the mixture was manually shaken for 10 min. The tubes were centrifuged at 4000 × *g* for 7 min, and 1.5 mL of the supernatant were transferred to a 2 mL polypropylene tube containing 200 mg of magnesium sulfate, 50 mg of PSA, and 50 mg of C_18_. The mixture was vortexed for 5 min and centrifuged under the same conditions.

The extraction procedure for glyphosate and AMPA in sesame grains was adapted from Martins-Júnior et al.^18^ Ground samples were homogenized, and a 2.0 g sample was weighed into 50 mL polypropylene tubes. Then, 10.0 mL of ultrapure water and 5.0 mL of dichloromethane were added, followed by mechanical shaking on a horizontal shaker (Tecnal TE-1401, Piracicaba, Brazil) at 250 rpm for 3 h. After shaking, the samples were centrifuged at 1350 × *g* for 10 min at 4 °C, and a 1.0 mL aliquot of the supernatant was transferred to a 2 mL polypropylene tube containing 1.0 mL of methanol. The tube contents were vortexed for 1 min and centrifuged at 4100 × *g* for 10 min at 4 °C.

Finally, after each extraction, 1.0 mL of the resulting extract from all samples was filtered through a 0.22 μm PTFE syringe filter to remove particulates and then transferred to a chromatography vial for subsequent LC-MS/MS analysis. The injection volumes were 25 μL for diquat, glyphosate, and AMPA, and 10 μL for saflufenacil.

### 2.7 Statistical Data Analysis

All quantitative data obtained from the LC-MS/MS analyses were converted to mg kg^–1^ considering the sesame sample mass used in the extraction procedure and the final volume of extraction solution obtained after sample preparation. This conversion allowed comparison with the MRLs established by national and international regulations.

The data obtained was subjected to analysis of variance (ANOVA) using RStudio software (version 4.4.1, Boston, MA, USA). Prior to ANOVA, normality and homogeneity of variance assumptions were evaluated using the Shapiro–Wilk test. When significant effects were detected, means were compared using Tukey’s test (*p* < 0.05).

## 3 Results

### 3.1 LC-MS/MS Method Validation

The chromatographic method was highly selective for all herbicides, showing clear separation of the analytes and no evidence of peak overlap or matrix interference at their respective Rt. Chromatographic elution resulted in Rt of 1.8 min for diquat, 1.02 min for saflufenacil, 0.5 min for AMPA, and 1.9 min for glyphosate (Figure 1). The calibration curve for all herbicides showed excellent linearity, with an R^2^ ≥ 0.97 for diquat, and ≥0.99 for saflufenacil, glyphosate, and AMPA (Figure 2).

**Figure 1:**
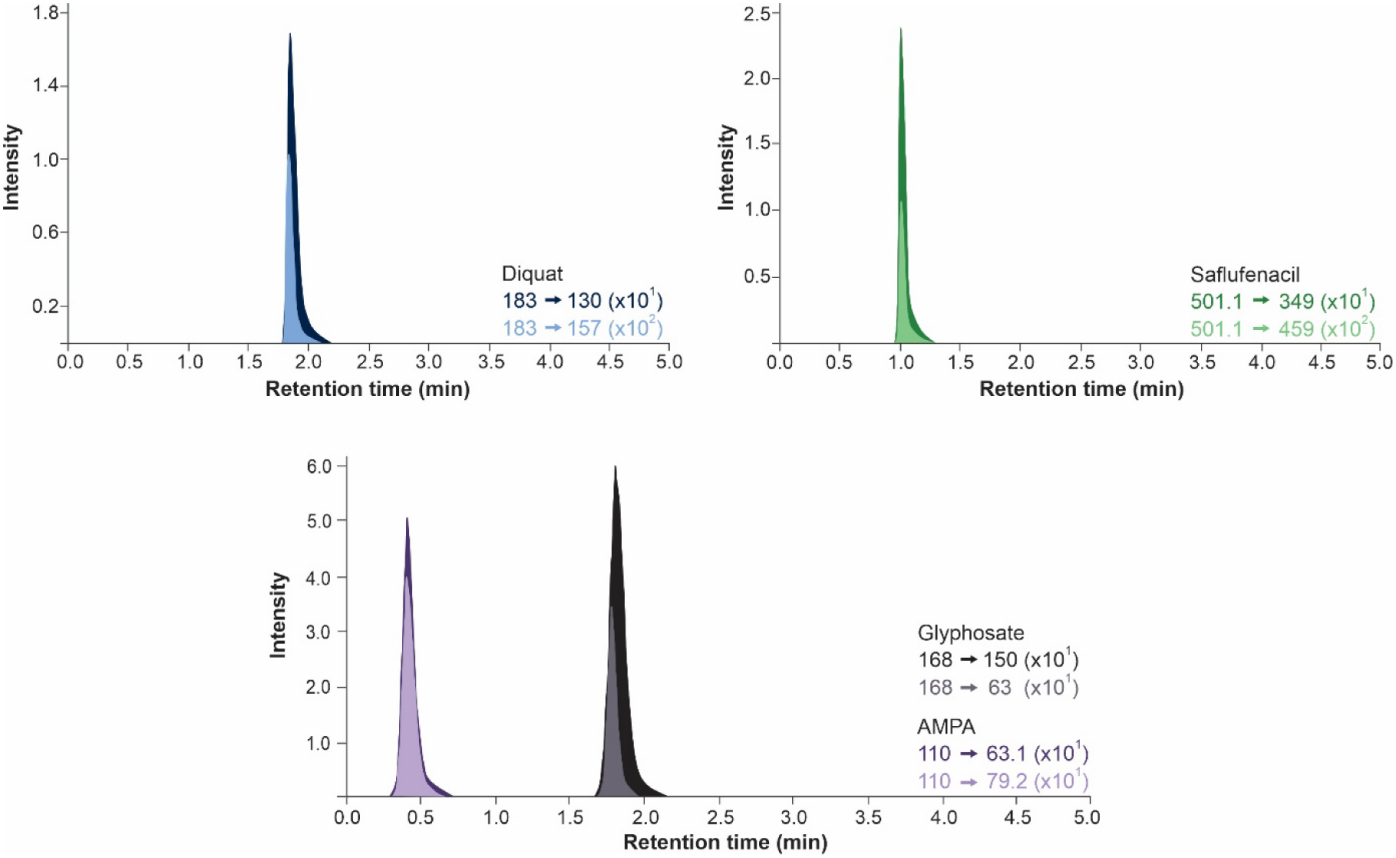
Chromatograms of diquat, saflufenacil, glyphosate, and its metabolite AMPA were detected by LC-MS/MS. with retention times (Rt) of 1.8 min for diquat, 1.02 min for saflufenacil, 0.5 in for AMPA, and 1.9 min for glyphosate. Total run time was 5 min.

**Figure 2:**
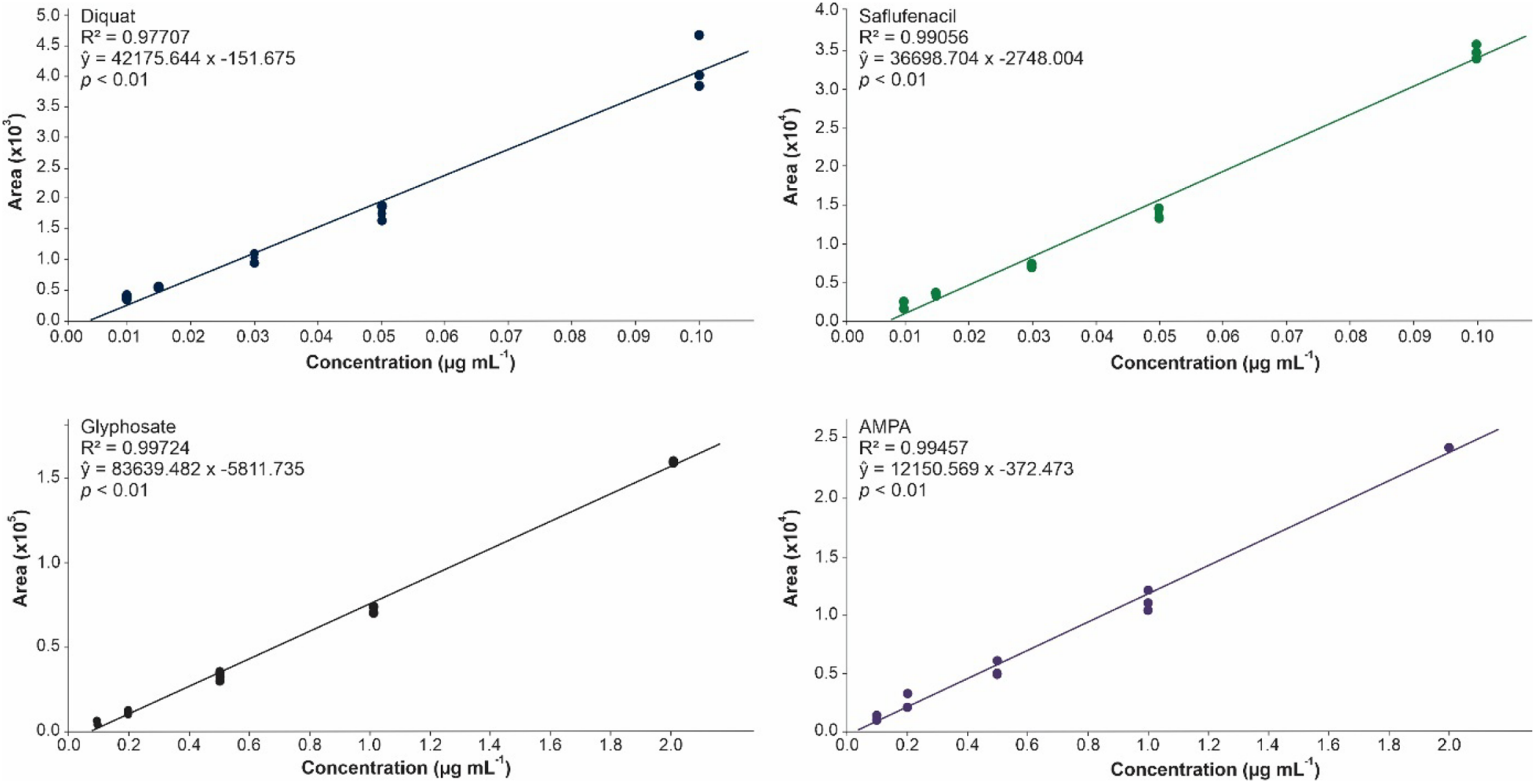
Calibration curves of the herbicides diquat, saflufenacil, and glyphosate, and the metabolite AMPA in sesame grains. Calibration levels were established at 0.01, 0.015, 0.03, 0.05, and 0.10 μg mL^–1^ for diquat and saflufenacil, and at 0.10, 0.20, 0.50, 1.00, and 2.00 μg mL^–1^ for glyphosate and AMPA. Each calibration point represents one concentration level, analyzed in triplicate.

The accuracy of the method, expressed as recovery percentages, demonstrated satisfactory performance for all analytes. The recoveries ranged from 91.7% to 111.5% for diquat, from 72.7% to 96.9% for saflufenacil, from 77.1% to 94.2% for glyphosate, and from 77.3% to 87.5% for AMPA, indicating that the extraction and quantification procedures were accurate across the tested concentration levels. The chromatographic method also exhibited good precision, with RSD values of 8.6% for diquat, 9.9% for saflufenacil, 7.3% for glyphosate, and 4.6% for AMPA.

The method exhibited high sensitivity, with LoD and LoQ values of 0.005 and 0.01 μg mL^–1^ for diquat, 0.006 and 0.02 μg mL^–1^ for saflufenacil, 0.03 and 0.10 μg mL^–1^ for glyphosate, and 0.07 and 0.22 μg mL^–1^ for AMPA, respectively.

### 3.2 Desiccation of Sesame Plants

Sesame plants exhibited rapid growth and uniform development under greenhouse conditions, with all individuals presenting similar phenological stages at the time of herbicide spraying. Plants treated with saflufenacil showed mild desiccation symptoms, characterized mainly by leaf abscission and partial capsule drying, even after 20 days following application (Figure S1). Many pods remained green even at harvest, requiring subsequent drying in an oven to complete the process.

Plants desiccated with diquat began showing characteristic symptoms of this herbicide’s mode of action (MoA) on the same day of spraying, including visible necrosis on leaves and pods. In areas where spray coverage was insufficient, symptoms were absent in the first few days; however, by harvest, even plants exposed to the lowest dose became completely desiccated. Moreover, in plants receiving the highest doses, several seeds exhibited a “burned” appearance, reflecting the stronger injury effect of the herbicide on the pods (Figure 3).

**Figure 3:**
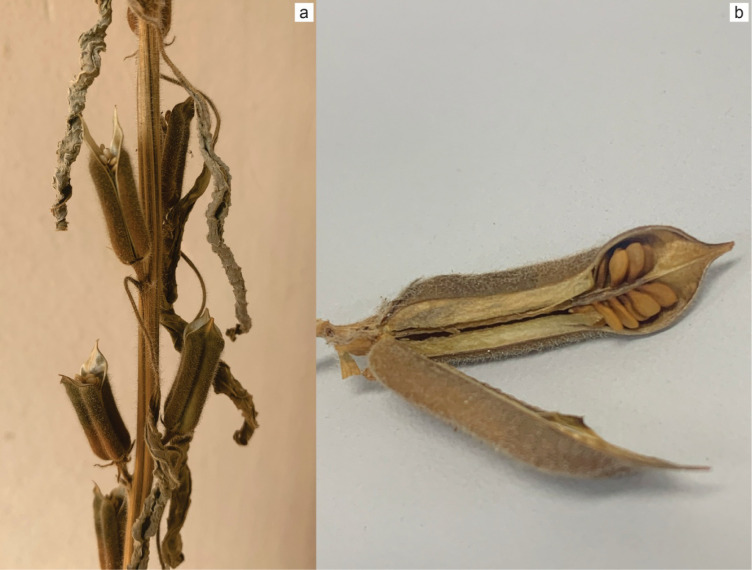
Appearance of sesame plants desiccated with diquat at all spraying doses (200, 300, and 400 g a.e. ha^–1^) at harvest (a) and pods showing “burned” seeds (b).

Plants treated with glyphosate began to show visible symptoms 1 week after application, with chlorosis starting at the lower leaves, while the pods remained green. The yellowing gradually progressed from the lower to the upper parts of the plant, and the intensity of these effects increased with the applied dose. The desiccation process was relatively slow at lower doses, where complete drying was not achieved by harvest time, with only a few pods showing slight burn symptoms (Figure S2).

Overall, visual evaluations demonstrated that only diquat provided rapid and complete desiccation of the sesame plants and pods, making it agronomically viable for anticipating harvest. In contrast, both saflufenacil and glyphosate failed to achieve adequate and uniform plant drying within the evaluated time frame.

### 3.3 Desiccant Residues in Sesame Seeds

Following the completion of the method validation and residue extraction procedures, chromatographic analyses were performed to quantify the desiccant residues for each herbicide in sesame seeds. For diquat and saflufenacil, residue levels remained below the respective LoQ values of 0.03 and 0.12 mg kg^–1^ in all samples, with only background noise observed in the chromatograms.

In contrast, the analysis of sesame seeds treated with different glyphosate doses revealed high levels of herbicide residues in all samples (Table 2). The lowest applied dose resulted in an average glyphosate concentration of approximately 26.9 mg kg^–1^, while the intermediate dose produced a lower residue level of 23.6 mg kg^–1^. The highest dose led to the greatest accumulation, with 51.7 mg kg^–1^ of glyphosate detected in the seeds. Statistically, the highest dose (960 g a.e. ha^–1^) differed significantly from the lower doses (480 and 720 g a.e. ha^–1^). Regarding the main glyphosate metabolite, AMPA, its concentrations remained below the LoQ (2.2 mg kg^–1^) in all analyzed samples, indicating that AMPA concentrations, if present, remained below the method quantification limit under the experimental conditions.

**Table 2:** Residues of Glyphosate and Its Metabolite AMPA in Sesame Seeds Sprayed with Different Glyphosate Doses

Treatment	Glyphosate (mg kg^–1^)^a^	AMPA (mg kg^–1^)^b^
Glyphosate 480 g a.e. ha^–1^	26.9 ± 1.4 b	<LoQ
Glyphosate 720 g a.e. ha^–1^	23.6 ± 2.6 b	<LoQ
Glyphosate 960 g a.e. ha^–1^	51.7 ± 5.2 a	<LoQ

a Values within parentheses indicate the standard deviation (±SD) of the results (*n* = 4). Means followed by the same letter in the column do not differ significantly (Tukey’s test at *p* < 0.05).

b LoQ of AMPA = 2.2 mg kg^–1^.

## 4 Discussion

### 4.1 LC-MS/MS Method Validation

The chromatographic and extraction procedures developed in this study provided adequate selectivity, accuracy, precision, and sensitivity for the determination of diquat, saflufenacil, glyphosate, and AMPA residues in sesame seeds. The analytical performance met the validation criteria established by Anvisa^23^ ans Inmetro^24^ confirming the reliability and robustness of the methods, with recoveries ranging between 70 and 120% and RSD values ≤20%. These results validate that the developed methods are appropriate for the precise quantification of herbicide residues in sesame matrices and are also in line with the European SANTE/11312/2021 guideline.^19^

Comparable analytical performance has been reported by other authors developing multiresidue methods for sesame and other oilseed matrices.^11,20,21^ These validation results obtained in this study are in line with those reported in the literature, supporting the reliability and analytical robustness of the proposed method for routine monitoring of herbicide residues in sesame and related matrices. Overall, the LC-MS/MS methods developed in this study proved to be reliable for the determination of diquat, saflufenacil, glyphosate, and AMPA residues in sesame seeds.

### 4.2 Desiccation of Sesame Plants

The evaluated desiccants exhibited markedly different desiccation efficiencies in sesame plants. Saflufenacil showed limited effectiveness, with most capsules remaining green even at the highest dose, suggesting restricted herbicide activity under the experimental conditions. Although protoporphyrinogen oxidase (PPO) inhibitors typically cause rapid necrosis of exposed tissues,^22,23^ such symptoms were not clearly observed in this study, indicating that factors such as application coverage, plant developmental stage, or limited herbicide translocation may have reduced its effectiveness.

In contrast, diquat induced rapid necrosis of leaves and pods, consistent with the typical response associated with contact herbicides.^22,24^ However, at higher doses, several seeds exhibited a burned appearance, suggesting that the rapid tissue desiccation caused by diquat may also have affected pod integrity and seed quality.

Glyphosate treatments resulted in a slower desiccation process, with symptoms appearing approximately 1 week after application and progressing gradually from lower to upper plant tissues. This delayed response is consistent with the systemic mode of action of glyphosate and its reliance on metabolic disruption rather than immediate cellular damage.^23,25,26^ As a consequence, lower doses were insufficient to promote complete plant drying before harvest.

The results indicate that the evaluated desiccants differ substantially in their desiccation dynamics in sesame plants. Importantly, since this study was conducted under controlled greenhouse conditions with potted plants, factors such as restricted root volume and altered plant architecture may differ from those observed under field conditions, potentially affecting desiccation efficiency and residue accumulation. Therefore, these findings should be considered as a preliminary greenhouse evaluation, highlighting the need for further studies to determine appropriate application strategies and validate agronomic efficacies under field conditions.

### 4.3 Desiccant Residues in Sesame Seeds

Across all treatments glyphosate residue levels exceed the MRLs established by Anvisa (10 mg kg^–1^),^27^ the European Union (0.1 mg kg^–1^),^28^ highlighting a potential risk for international trade compliance. The Codex Alimentarius has not established specific MRLs for glyphosate or the other evaluated herbicides in sesame, which further reinforces the regulatory uncertainty and potential risks for international trade compliance.^29^ In the absence of crop-specific MRLs, some regulatory agencies adopt default tolerance limits for unregistered pesticides, which may vary from 0.01 to 0.1 mg kg^–1^.^30^ The European Food Safety Authority (EFSA) is reviewing the current MRLs for glyphosate in the European Union.^31^

Glyphosate is a highly mobile systemic herbicide that translocates through the phloem.^25,26^ When applied at advanced reproductive stages, the developing capsules and seeds become strong metabolic sinks, promoting the translocation and accumulation of glyphosate in reproductive tissues, a phenomenon often described as a “diversion of translocation” toward the seeds.^32^ This behavior helps explain the high residue levels detected in sesame grains at harvest.

In contrast to glyphosate, saflufenacil and diquat residues remained below the LoQ (0.12 mg kg^–1^, 0.03 mg kg^–1^, respectively). This striking difference in residue accumulation is likely associated with the distinct mobility and translocation characteristics of each herbicide within the plant. Diquat and saflufenacil are contact herbicides and cause rapid cellular disruption followed by tissue necrosis,^23–25^ with symptoms manifest through localized necrotic spots, limiting the effect only to the areas hit by the droplets.^33^ Because the seeds are physically protected inside the capsules, they do not come into direct contact with the spray droplets, explaining the complete absence of diquat and saflufenacil residues inside the analyzed grains.

Regarding AMPA, concentrations were below the LoQ (<2.2 mg kg^–1^) across all treatments. However, this result must be interpreted cautiously due to analytical constraints. Although glyphosate metabolism in plants is considered limited,^34^ AMPA has still been detected in several food matrices, including cereals, wheat-derived foods, soybean products, pulses, and processed foods,^14^ particularly following preharvest applications.^35^

The determination of AMPA is analytically challenging given its high polarity and water solubility.^36,37^ Thus, the relatively high LoQ achieved in this study may have limited the detection of trace AMPA concentrations and does not necessarily indicate the absolute absence of this metabolite in sesame seeds. Considering that AMPA exhibits toxicity comparable to glyphosate,^38^ the use of more sensitive analytical approaches in future studies is recommended to better characterize its occurrence and accumulation in sesame crops.

These findings indicate that glyphosate residues persist in sesame seeds at harvest, raising concerns about food safety and regulatory compliance. The detection of such concentrations are particularly critical given previous studies reporting pesticide residues, such as lindane, chlorpyrifos, and metalaxyl, not only in sesame seeds but also in sesame oil, demonstrating that residues may be transferred to processed products.^39^

Noncompliance with MRLs can have severe trade implications. The European Union has established strict regulatory (EU 2020/1540) for pesticide residues in sesame seeds, including mandatory compliance with MRL legislation and official certification for imported products, reinforcing the importance of continuous residue monitoring to ensure food safety and international trade compliance.^40^

This case highlights the importance of establishing and monitoring pesticide residues in Brazilian sesame production to ensure export compliance and consumer safety. Given the growing international demand for sesame and the limited number of studies on herbicide residues, further research is needed to develop strategies that balance desiccation efficiency, grain quality, and residue reduction.

In addition, further studies are essential to support the registration and regulation of desiccant herbicides in sesame cultivation. Although desiccation practices are already used by producers, they are often performed empirically and without crop-specific technical recommendations, increasing the risk of inadequate applications, elevated residue levels, and noncompliance with international food safety standards. Establishing appropriate application strategies, preharvest intervals, and residue limits for sesame is therefore critical to support safe production practices and strengthen access to international markets.

## Supplementary Material



## Notes

Physicochemical properties of the soil used in this study, Piracicaba, São Paulo, Brazil, 2025 (Table S1); appearance of sesame plants at harvest following desiccation with saflufenacil at 35 (a), 52.5 (b), and 70 g a.i. ha^–1^ (c) doses (Figure S1); appearance of sesame plants at harvest following desiccation with glyphosate at 480 (a), 720 (b), and 960 g a.e. ha^–1^ (c) doses (Figure S2) (PDF)

## Abbreviations

PHIs: preharvest intervals
MRLs: maximum residue limits
AMPA: aminomethylphosphonic acid
LC-MS/MS: liquid chromatography coupled to tandem mass spectrometry
DAA: days after application
ESI: electrospray ionization
MRM: multiple reaction monitoring
LoD: limits of detection
LoQ: limits of quantification
Rt: retention times
RSD: relative standard deviation
d-SPE: dispersive solid-phase extraction
PSA: primary secondary amine
PTFE: polytetrafluoroethylene
ANOVA: analysis of variance
MoA: mode of action
PPO: protoporphyrinogen oxidase
EFSA: European Food Safety Authority
