# Supplementary material for: Residues of Desiccant Herbicides in Sesame Seeds (Sesamum indicum L.) Following Preharvest Application Determined by LC-MS/MS
Source: J Agric Food Chem. 2026 Jul 1;74(27):21515–22. doi: 10.1021/acs.jafc.6c06511 (PMC13383727; doi:10.1021/acs.jafc.6c06511)
Supplement: Supplementary file 1 [file jf6c06511_si_005.pdf]

Supporting Information

Residues of desiccant herbicides in sesame seeds (*Sesamum indicum* L.) following pre-harvest application determined by LC–MS/MS

Laura Bordignon*[1], Rodrigo Monte Lorenzoni[2], Thiago Svacina[1], Rodrigo Floriano Pimpinato[1], Kassio Ferreira Mendes[1]

[1] Center for Nuclear Energy in Agriculture, University of São Paulo, Centenário Avenue, 303, São Dimas, Piracicaba, 13400-970, São Paulo, Brazil;

[2] Sebra Agrícola LTDA, Research and Development Department, Lot. Canarana I, Chácara G8, Canarana, 78640-000, Mato Grosso, Brazil;

Corresponding author address: laurabrdgn@usp.br

Table of contents

Table S1. Physicochemical properties of the soil used in this study, Piracicaba, São Paulo, Brazil, 2025.. S2

Figure S1. Appearance of sesame plants at harvest following the desiccation with saflufenacil at 35 (a), 52.5 (b), and 70 g a.i. ha^{-1} (c) doses................................. S3

Figure S2. Appearance of sesame plants at harvest following the desiccation with glyphosate at 480 (a), 720 (b), and 960 g a.e. ha^{-1} (c) doses............................... S4

Table S1**.** Physicochemical properties of the soil used in this study, Piracicaba, São Paulo, Brazil, 2025.

Al^{3+}	$H^+ + Al^{3+}$	Mg^{2+}	Ca^{2+}	K^+
		$mmol_c\ dm^{-3}$		
0.0	28.3	25.4	69.3	9.4

B	Cu	Fe	Zn	Mn	P
		$mg\ dm^{-3}$			
0.42	5.4	27.2	4.0	85.4	51.6

pH	OC	CEC	BS	Clay	Silt	Sand
$CaCl_2$	%	$mmol_c\ dm^{-3}$	%		%	
5.63	3.17	132.3	79.0	43.3	21.0	35.7

pH: hydrogen ion potential; OC: Organic Carbon content; CEC: Cation Exchange Capacity. BS: base saturation. Source: Soil Analysis Laboratory, Department of Soil Sciences, ESALQ/USP, Piracicaba, São Paulo, Brazil.

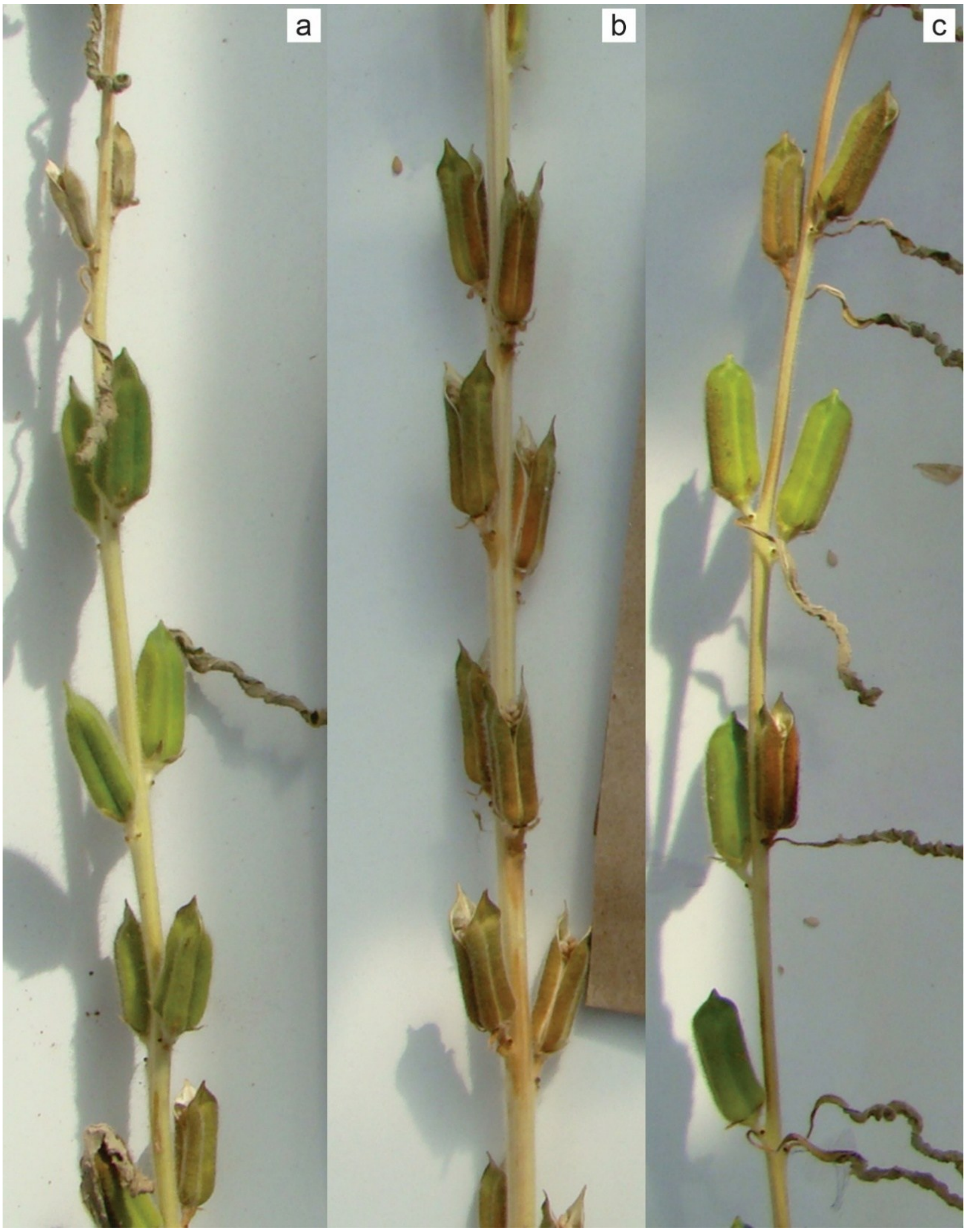

Figure S1. Appearance of sesame plants at harvest following the desiccation with saflufenacil at 35 (a), 52.5 (b), and 70 g a.i. ha^{-1} (c) doses.

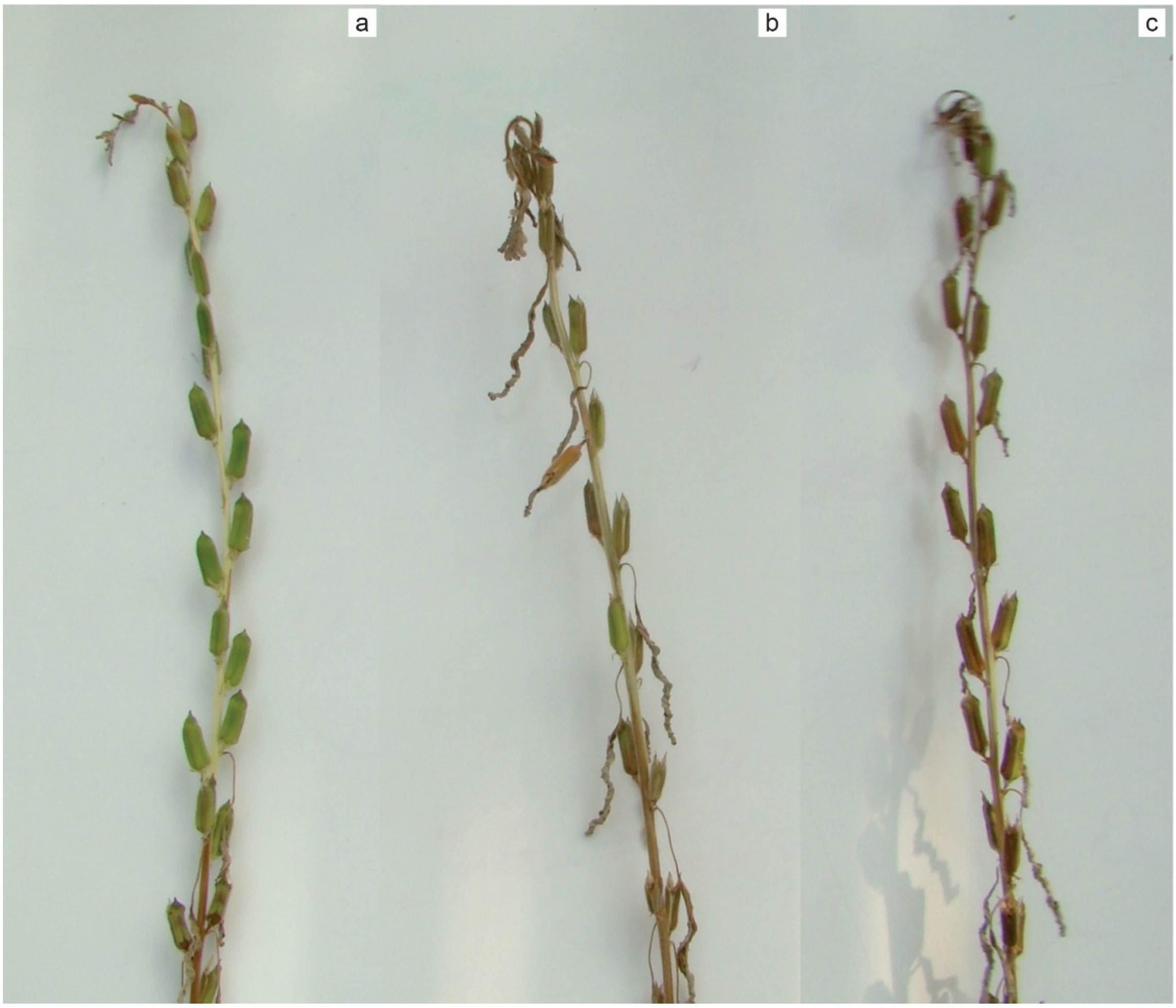

Figure S2. Appearance of sesame plants at harvest following the desiccation with glyphosate at 480 (a), 720 (b), and 960 g a.e. ha^{-1} (c) doses.
